# Perceptions of medical students at Imam Mohammad Ibn Saud Islamic University on histology’s role in clinical course preparation: A cross-sectional study

**DOI:** 10.1371/journal.pone.0337894

**Published:** 2025-12-05

**Authors:** Eman Ali Elkordy, Mohammed Sulaiman Alwohaibi, Yazeed Musa AlHarbi, Abdulrahman Mohammed Alanazi, Hessah Abdulrahman Almojel, Bandar Nawaf Altimyat, Mohammad Ibrahim Jumaa, Safaa Masoud Hanafy

**Affiliations:** 1 Anatomy and physiology Department, College of Medicine, Imam Mohammad Ibn Saud Islamic University (IMSIU), Riyadh, Saudi Arabia; 2 Medical student, College of Medicine, Imam Mohammad Ibn Saud Islamic University (IMSIU), Riyadh, Saudi Arabia; AIIMS: All India Institute of Medical Sciences, INDIA

## Abstract

**Background:**

Histology is a foundational discipline in medical education that connects basic biomedical sciences with clinical practice. However, its perceived relevance may diminish as students progress academically, especially when integration with clinical teaching is limited. This study aimed to assess medical students’ perceptions of histology’s value, teaching quality, and clinical integration at Imam Mohammad Ibn Saud Islamic University across different academic levels.

**Methods:**

A cross-sectional survey was conducted in January 2025 among 235 undergraduate medical students. A validated, self-administered questionnaire measured students’ perceptions of histology’s importance, teaching effectiveness, clinical relevance, curricular integration, and overall satisfaction. Quantitative data were analyzed using descriptive statistics, ANOVA, logistic regression, and Pearson correlation.

**Results:**

Most students rated histology as important or very important (73.6%) and acknowledged its contribution to understanding disease mechanisms (88.5%). Satisfaction was strongly associated with perceived applicability, visualization, and integration. Lectures and laboratory sessions were rated as effective or very effective by 75.7% and 76.2% of students, respectively. Additionally, 80.9% supported stronger clinical integration, and 83% preferred the inclusion of clinical examples. Perceptions varied across academic stages, with preclinical students expressing more favorable views. Regression analysis identified teaching quality and integration with clinical content as significant predictors of positive perceptions.

**Conclusions:**

Histology continues to be a valued component of medical education when delivered through effective teaching and clinically relevant integration. Strengthening instructional design and embedding clinical context throughout the curriculum may enhance student engagement, improve learning outcomes, and sustain the relevance of histology across all stages of medical training.

## Introduction

Histology, the microscopic study of tissue structure, is a foundational component of undergraduate medical education. It provides students with essential knowledge about the organization and function of cells, tissues, and organs, serving as a critical bridge between basic biomedical sciences and clinical practice. A firm understanding of histological concepts enhances students’ abilities to interpret pathological changes and supports diagnostic reasoning in various medical specialties, particularly pathology, dermatology, and oncology [1–4].

The teaching of histology, characterized by its reliance on image-based content, remains one of the most challenging aspects of medical education. A variety of pedagogical strategies, encompassing both traditional and nontraditional methods have been employed to enhance learning outcomes. These include peer-assisted learning, audiovisual aids, and both technological and manual drawing techniques, all of which have been systematically investigated. In addition, virtual microscopy has demonstrated significant pedagogical benefits [5,6].

Several studies have highlighted the critical role of histology in fostering clinical reasoning and diagnostic accuracy among medical students. By facilitating an understanding of disease processes at the cellular and tissue levels, histology provides a critical framework for interpreting pathological alterations in clinical contexts. It was found that students who perceived strong integration of histology and pathology reported greater confidence in interpreting clinical cases. Similarly, integrating virtual microscopy with clinical cases was found to improve students’ ability to correlate morphological features with clinical reasoning, thereby supporting the earlier development of diagnostic competence. These findings align with broader educational trends that advocate for integrating basic sciences with clinical teaching to enhance relevance, retention, and application of knowledge in clinical settings [7–9].

Despite its acknowledged importance, questions remain regarding how effectively students can apply the advanced knowledge gained from histology and other basic sciences within their clinical training. Historically, medical education has placed more emphasis on practical aspects such as tissue preparation and interpreting histological slides, often in isolation from clinical application. However, with the advent of integrated and competency-based curricula, there is increasing interest in whether students perceive histology as a relevant and practically applicable subject in clinical decision-making. This shift has prompted educators to reevaluate how histology is taught, moving beyond rote identification of tissues toward emphasizing its clinical correlations. The ability to translate microscopic knowledge into diagnostic reasoning and patient care is now seen as a key objective of modern medical education. Consequently, understanding students’ perceptions of histology’s clinical relevance is essential for guiding curriculum design and aligning teaching methods with real world clinical competencies [10–13].

Furthermore, despite its recognized importance, there has been growing concern that the perceived relevance of histology may diminish as students progress through medical school. This phenomenon is often attributed to limited integration of histological knowledge into clinical teaching and insufficient contextualization during clinical rotations. As medical education increasingly emphasizes problem-based and integrated curricula, understanding how students perceive the value, relevance, and application of histology is essential for optimizing instructional strategies and promoting deeper learning. Without explicit reinforcement in clinical settings, students may fail to connect basic histological principles with disease mechanisms and diagnostic processes. This disconnect can lead to underutilization of foundational knowledge in clinical reasoning, ultimately affecting the quality of diagnostic accuracy and decision-making. Investigating students’ evolving perceptions can thus provide crucial feedback for curriculum development aimed at maintaining the clinical salience of histology throughout all stages of medical training [5,6,12,14–16].

Previous research has highlighted that effective teaching methods, such as active learning, visual aids, and clinical correlation, are key to fostering student engagement and improving learning outcomes in histology. These pedagogical approaches not only enhance conceptual understanding but also facilitate the retention and clinical application of histological knowledge. Despite this, empirical data on how students at different academic levels evaluate their histology education, particularly in terms of teaching quality, integration, and clinical applicability remain limited, especially within the context of Saudi Arabian medical schools. Such contextual data are essential for identifying whether current teaching practices meet the learning needs of students at various stages of their medical education. Understanding these perceptions can inform targeted interventions to enhance the design, delivery, and integration of histology education, ensuring that it remains a clinically meaningful and educationally effective component of the medical curriculum [17–19].

While several international studies have examined students’ perceptions of histology education, there is limited literature exploring this topic within the context of Saudi Arabia and the broader Middle East. Most existing research has been conducted in Western or Asian academic settings, where curricular structures and educational priorities may differ substantially. This study adds a novel perspective by providing localized data from a Saudi institution implementing an integrated, outcome-based curriculum. Moreover, it explores differences in perception across academic stages and employs multivariate analysis to identify predictors of positive attitudes, offering a more nuanced understanding of students’ learning experiences. These contributions address a regional evidence gap and offer practical insights that may inform histology education reforms in comparable educational environments.

The present study aims to evaluate the perceptions of medical students at Imam Mohammad Ibn Saud Islamic University regarding the role of histology in their clinical course preparation. It investigates their views on the subject’s relevance, integration with clinical sciences, and educational effectiveness across different academic levels. Such insights can inform targeted curricular interventions to improve educational outcomes and ensure that histology remains a clinically relevant component of medical training.

## Materials and methods

### Study setting and design

This cross-sectional study was conducted in January 2025 at Imam Mohammad Ibn Saud Islamic University, Saudi Arabia. The study aimed to assess medical students’ perceptions regarding the role of histology in preparing them for clinical courses and its impact on their clinical reasoning skills. Data were collated between 01/01/2025 and 31/01/2025.

### Sampling procedure and study population

The target population included undergraduate medical students (≥18 years old) enrolled across all academic years at Imam Mohammad Ibn Saud Islamic University. A convenience sampling method was employed to recruit participants from different academic years. While the sample was not probabilistic, efforts were made to include a wide range of students across pre-clinical and clinical levels to enhance diversity and minimize sampling bias within the limitations of voluntary participation. This approach was selected due to practical constraints related to accessibility and timing. Eligible students who had completed or were currently enrolled in histology courses were invited to participate voluntarily. No minors were included in the study; therefore, parental or guardian consent was not required.

### Inclusion and exclusion criteria

Participants were included in this study if they met the following criteria: (1) were 18 years of age or older, (2) were currently enrolled as medical students at Imam Mohammad Ibn Saud Islamic University, and (3) provided voluntary informed consent. Exclusion criteria included: (1) being younger than 18 years of age, (2) having graduated from the program, (3) declined to provide informed consent. All participants were 18 years or older; therefore, consent from parents or guardians was not required.

### Sample size calculation

The sample size was calculated based on a 95% confidence level, a standard deviation of 0.5, and a 5% margin of error, yielding a minimum required sample of 385 participants. To accommodate potential non-responses or incomplete data, a target of 400 participants was set. Ultimately, 235 students completed the survey, resulting in a response rate of 59%.

### Data collection instrument

A structured, self-administered questionnaire was developed and distributed via Google Forms. The instrument was reviewed by subject experts for face validity and clarity and pilot-tested with a small group of students (n = 15) to ensure reliability and relevance. It consisted of both closed- and open-ended questions, divided into four sections:

- Section 1: Demographic data (age, gender, academic year).
- Section 2: Perceptions of the value and importance of histology using Likert-scale items.
- Section 3: Views on the integration of histology with clinical subjects.
- Section 4: Confidence in applying histological knowledge in clinical settings.

### Questionnaire reliability and validity

The questionnaire underwent expert review to ensure **content and face validity**, with three faculty members in histology and medical education evaluating each item for clarity, relevance, and comprehensiveness. A pilot study involving 15 students was conducted to test item reliability and consistency. Internal consistency of the final questionnaire was assessed using Cronbach’s alpha, yielding a value of **0.83**, indicating good reliability. Minor wording adjustments were made based on pilot feedback before large-scale distribution.

### Data collection procedure

Data collection was conducted over a four-week period in January 2025. An invitation email containing the survey link and study information was distributed via official university email addresses. A reminder email was sent two weeks later to maximize response rates. The survey began with an introduction explaining the study’s purpose and significance. Informed consent was obtained electronically before accessing the questionnaire. Participation was voluntary and anonymous. The average completion time was 10–15 minutes.

### Questionnaire scoring

To quantitatively assess students’ perceptions across key domains, four composite scores were developed based on responses to Likert-scale items. All items were reverse-coded where necessary to ensure that higher scores consistently reflected more favorable evaluations.

- The Perception of Value Score measured students’ overall views on the importance and clinical relevance of histology. It was calculated as the mean of four items assessing the perceived role of histology in clinical preparation, relevance to clinical coursework, contribution to understanding disease mechanisms, and applicability to future clinical practice.
- The Teaching Effectiveness Score reflected students’ evaluations of the quality of histology instruction, including both lectures and laboratory sessions. Items were averaged after reverse coding, with higher values indicating more positive assessments.
- The Integration Quality Score captured students’ perceptions of how effectively histology content was integrated with clinical subjects.
- The Overall Experience Score assessed students’ satisfaction with the histology course, particularly in terms of how well it supported the visualization of tissues and organs in a clinical context. This score was derived by averaging two reverse-coded items.

### Likert scale description

Responses to perception items were measured using a five-point Likert scale, where 1 = strongly disagree, 2 = disagree, 3 = neutral, 4 = agree, and 5 = strongly agree. Higher scores indicated more favorable perceptions of histology’s importance, teaching quality, and curricular integration. For composite score calculations, all negatively worded items were reverse-coded to ensure that higher mean scores consistently reflected more positive evaluations.

### Data analysis

Quantitative data were analyzed using SPSS version 29 and R version 4.3.0.

SPSS was used to perform descriptive statistics (means, standard deviations, frequencies, and percentages) and Pearson correlation analyses to examine relationships among perception domains. R was used to conduct inferential statistical analyses, including Kruskal–Wallis tests for comparing medians across academic stages, Fisher’s exact tests for categorical variables with small cell sizes, and multivariate logistic regression to identify predictors of positive perceptions while adjusting for gender, academic year, and histology course completion.

Data distribution was assessed using the Shapiro–Wilk test, which indicated that most perception variables (Likert-scale items) were non-normally distributed; therefore, non-parametric tests were applied for group comparisons. A significance threshold of *p* < 0.05 was applied throughout. Missing data were handled using pairwise deletion for descriptive analyses and listwise deletion for regression models.

### Ethics statement

Ethical approval for this study was obtained from the Institutional Review Board (IRB) of the College of Medicine, Imam Mohammad Ibn Saud Islamic University (Approval No. HAPO-01-R-011, dated December 22, 2024). All participants were 18 years or older and provided informed consent electronically prior to survey participation.

## Results

Table 1 summarizes the demographic and academic characteristics of the study sample (N = 235). The mean age of participants was 21.7 years (SD = 1.3). The majority of participants were male (71.9%, n = 169), while females comprised 28.1% (n = 66). Participants were distributed across all years of medical study, with relatively balanced representation from Years 1–4. Specifically, 26.8% (n = 63) were in Year 1, 25.1% (n = 59) in Year 2, 16.6% (n = 39) in Year 3, and 27.7% (n = 65) in Year 4. A smaller proportion were Year 5 students (3.0%, n = 7) or interns (0.9%, n = 2). When categorized by academic stage, just over half of the respondents (51.9%, n = 122) were in the pre-clinical phase, while 44.3% (n = 104) were in the clinical phase, and 3.8% (n = 9) were in the advanced clinical phase. Regarding course exposure, 88.1% of participants (n = 207) had completed the histology course, whereas 11.9% (n = 28) had not.

**Table 1:** Descriptive characteristics of the study sample (N = 235).

Variable	Category	n (%)/ Mean ± SD
**Age**	Mean ± SD	21.7 ± 1.3
**Gender**	Female	66 (28.1%)
	Male	169 (71.9%)
**Year of study**	Year 1	63 (26.8%)
	Year 2	59 (25.1%)
	Year 3	39 (16.6%)
	Year 4	65 (27.7%)
	Year 5	7 (3.0%)
	Intern	2 (0.9%)
**Year group**	Pre-clinical	122 (51.9%)
	Clinical	104 (44.3%)
	Advanced clinical	9 (3.8%)
**Histology course completed**	Yes	207 (88.1%)
	No	28 (11.9%)

Note: Categorical variables are presented as frequencies and percentages. Missing data were addressed using pairwise deletion.

Table 2 presents a comprehensive overview of students’ perceptions regarding various dimensions of histology education. A large majority rated histology as either very important (38.3%) or important (35.3%), while only 9.0% perceived it as not important or not important at all. Regarding clinical relevance, most participants recognized a strong (47.2%) or very strong (32.8%) connection between histology and clinical courses, whereas only 0.9 reported no connection. In terms of clinical understanding, 50.6% indicated that histology helped significantly, while 37.9% reported it helped somewhat. A smaller proportion (11.5%) indicated that it was of limited or no help. On content relevance to clinical practice, 70.7% rated histology content as either very relevant or somewhat relevant, compared to 10.3% who found it not very or not at all relevant. Finally, overall satisfaction was favorable, with 70.2% expressing they were very satisfied or satisfied with the course, and only 8.9% indicating dissatisfaction.

**Table 2:** Medical students’ perceptions of histology: Importance, relevance, clinical impact, and satisfaction (N = 235).

Item	Response	n (%)
**Histology importance**	Very important	90 (38.3%)
	Important	83 (35.3%)
	Neutral	41 (17.4%)
	Not important	14 (6.0%)
	Not important at all	7 (3.0%)
**Histology–clinical relevance**	Very strong	77 (32.8%)
	Strong	111 (47.2%)
	Moderate	31 (13.2%)
	Weak	14 (6.0%)
	No connection	2 (0.9%)
**Histology aids clinical understanding**	Yes, significantly	119 (50.6%)
	Yes, somewhat	89 (37.9%)
	No, not really	19 (8.1%)
	No, not at all	8 (3.4%)
**Content relevance to clinical practice**	Very relevant	66 (28.1%)
	Somewhat relevant	100 (42.6%)
	Neutral	45 (19.1%)
	Not very relevant	18 (7.7%)
	Not relevant at all	6 (2.6%)
**Overall course satisfaction**	Very satisfied	70 (29.8%)
	Satisfied	95 (40.4%)
	Neutral	49 (20.9%)
	Dissatisfied	17 (7.2%)
	Very dissatisfied	4 (1.7%)

Frequencies and percentages are reported for categorical responses. Missing data were handled using pairwise deletion.

Table 3 presents students’ evaluations of histology teaching methods and their integration with clinical content. Lectures were viewed favorably, with 75.7% of students rating them as very effective (37.0%) or effective (38.7%). A small minority (5.1%) rated lectures as ineffective or very ineffective. Laboratory sessions received even more positive evaluations, with 76.2% describing them as very effective (48.5%) or effective (27.7%), although 11.0% perceived them as ineffective or very ineffective.

**Table 3:** Student ratings of histology teaching methods and integration with clinical courses (N = 235).

Item	Response	n (%)
**Effectiveness of lectures**	Very effective	87 (37.0%)
	Effective	91 (38.7%)
	Neutral	45 (19.1%)
	Ineffective	9 (3.8%)
	Very ineffective	3 (1.3%)
**Effectiveness of lab sessions**	Very effective	114 (48.5%)
	Effective	65 (27.7%)
	Neutral	30 (12.8%)
	Ineffective	13 (5.5%)
	Very ineffective	13 (5.5%)
**Integration with clinical courses**	Very well integrated	69 (29.4%)
	Well integrated	86 (36.6%)
	Neutral	60 (25.5%)
	Poorly integrated	19 (8.1%)
	Not integrated at all	1 (0.4%)
**Support for more integration**	Yes, absolutely	85 (36.2%)
	Yes, somewhat	105 (44.7%)
	No, remain separate	35 (14.9%)
	Not sure	10 (4.3%)
**Preference for clinical examples**	Yes, definitely	113 (48.1%)
	Yes, somewhat	82 (34.9%)
	No, prefer basic	34 (14.5%)
	Not sure	6 (2.6%)
**Helped visualize tissues**	Yes, very much	111 (47.2%)
	Yes, somewhat	89 (37.9%)
	Neutral	22 (9.4%)
	No, not really	9 (3.8%)
	No, not at all	4 (1.7%)

Regarding curricular integration, 66.0% of respondents indicated that histology was well integrated or very well integrated with clinical coursework, while only 8.5% rated it as poorly or not integrated. Support for enhanced integration was strong, with 80.9% expressing that histology should be better connected to clinical content, either absolutely (36.2%) or somewhat (44.7%).

When asked about instructional preferences, 83.0% of students favored the inclusion of more clinical examples, and only 14.5% preferred a focus on basic science alone. In terms of visual understanding, 85.1% reported that histology helped them very much or somewhat in visualizing tissues and organs. Additionally, 63.4% of participants voluntarily provided open-ended suggestions for improving histology instruction, indicating strong engagement with the subject matter.

**Fig 1:**
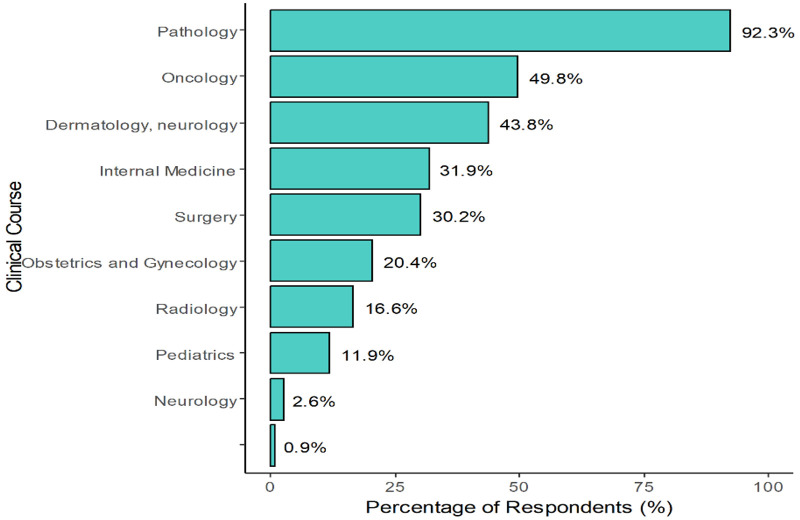
Clinical courses identified by students as benefiting from histology knowledge.

Note: Dermatology/Neurology’ and ‘Neurology’ were derived from open-ended responses to the ‘Other’ option in the questionnaire. These categories were not among the predefined choices and may reflect overlapping interpretations.

As illustrated in Fig 1, pathology was most frequently identified by respondents as the clinical discipline most enhanced by histology education, with 92.3% selecting it. This was followed by oncology (49.8%), dermatology/neurology (43.8%), and internal medicine (31.9%). Other disciplines such as surgery (30.2%), obstetrics and gynecology (20.4%), and radiology (16.6%) were also commonly cited. Less frequently selected were pediatrics (11.9%), neurology (2.6%), and a small number of other or unspecified areas (0.9%). These results suggest students perceive histology as particularly relevant to disease-focused specialties, especially those requiring strong microscopic diagnostic skills.

Table 4 presents a comparison of students’ perceptions of histology across academic stages: preclinical (n = 122), clinical (n = 104), and advanced clinical (n = 9). The data are expressed as median [interquartile range]. Significant differences were observed in several domains. The perceived importance of histology declined progressively with academic advancement (*p* < 0.001), with the highest perceived importance reported by preclinical students (median = 1.50 [1.00–2.00]) and the lowest by those in advanced clinical years (median = 3.00 [1.00–3.00]). Similarly, clinical relevance ratings were highest among clinical-year students (2.00 [2.00–2.25]) compared to their peers in preclinical and advanced groups (*p* = 0.010). Regarding histology’s role in aiding clinical understanding, preclinical students rated it most favorably (1.00 [1.00–2.00]), while clinical and advanced students provided slightly less favorable but still positive evaluations (*p* = 0.021). Perceptions of content relevance to clinical practice and integration with clinical courses increased across training levels (*p* = 0.003 and *p* < 0.001, respectively), indicating stronger perceived application of histological knowledge as students gained more clinical experience. No statistically significant differences were observed in students’ preference for clinical examples (*p *= 0.829), suggesting consistent support for clinical contextualization of histology across all academic levels. However, overall satisfaction with histology education was significantly higher among clinical and advanced groups compared to preclinical students (*p* < 0.001). These findings suggest that while appreciation of histology’s theoretical importance is stronger in early years, its perceived clinical applicability and integration become more evident as students gain clinical exposure, emphasizing the need for earlier contextual linkage in histology education.

**Table 4:** Comparison of perceptions toward histology across study years.

	Preclinical	Clinical	Advanced	p. overall
	N = 122	N = 104	N = 9	
**Histology Importance:**	1.50 [1.00;2.00]	2.00 [1.75;3.00]	3.00 [1.00;3.00]	<0.001
**Histology–Clinical Relevance:**	2.00 [1.00;2.00]	2.00 [2.00;2.25]	1.00 [1.00;3.00]	0.010
**Histology Aids Clinical Understanding:**	1.00 [1.00;2.00]	2.00 [1.00;2.00]	2.00 [2.00;2.00]	0.021
**Content Relevance to Clinical Practice:**	2.00 [1.00;2.00]	2.00 [2.00;3.00]	2.00 [1.00;3.00]	0.003
**Integration with Clinical Courses:**	2.00 [1.00;2.00]	2.00 [2.00;3.00]	3.00 [1.00;4.00]	<0.001
**Preference for Clinical Examples:**	2.00 [1.00;2.00]	2.00 [1.00;2.00]	2.00 [1.00;3.00]	0.829
**Overall satisfaction**	2.00 [1.00;2.00]	2.00 [2.00;3.00]	2.00 [1.00;3.00]	<0.001

Values are expressed as median [interquartile range]. Statistical comparisons were performed using the Kruskal-Wallis test. A *p*-value < 0.05 indicates a significant difference across groups. Academic groups were defined as follows: Preclinical = Years 1–2, Clinical = Years 3–4, Advanced Clinical = Years 5–intern.

**Fig 2:**
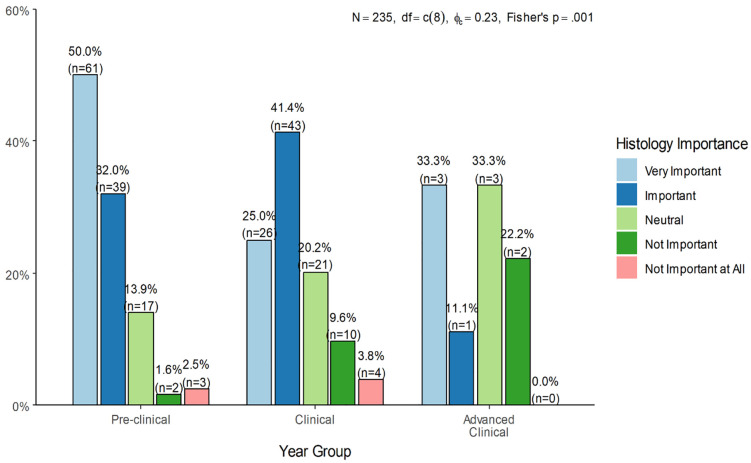
Perceived importance of histology by academic year group.

As illustrated in Fig 2, the perceived importance of histology varied significantly across academic stages (Fisher’s p = 0.001). Among pre-clinical students, the majority viewed histology as either very important (50.0%, n = 61) or important (32.0%, n = 39), with only 4.1% (n = 5) holding negative views (not important or not important at all). The clinical group exhibited a notable shift in their perception of histology’s importance compared to the pre-clinical group. While a substantial portion still considered histology important (41.4%, n = 43), only 25.0% (n = 26) rated it as very important. Additionally, there was a rise in neutral and negative responses within this group, with 9.6% (n = 10) selecting not important and 3.8% (n = 4) choosing not important at all. In the advanced clinical group, opinions were more divided: only 33.3% (n = 3) rated histology as very important, while 22.2% (n = 2) considered it not important and none selected not important at all. This pattern may reflect increased clinical demands and a perception that histology is less directly applicable or emphasized during clinical training. Nevertheless, the majority still viewed it positively, suggesting a recognized though possibly underutilized value in clinical education.

**Fig 3:**
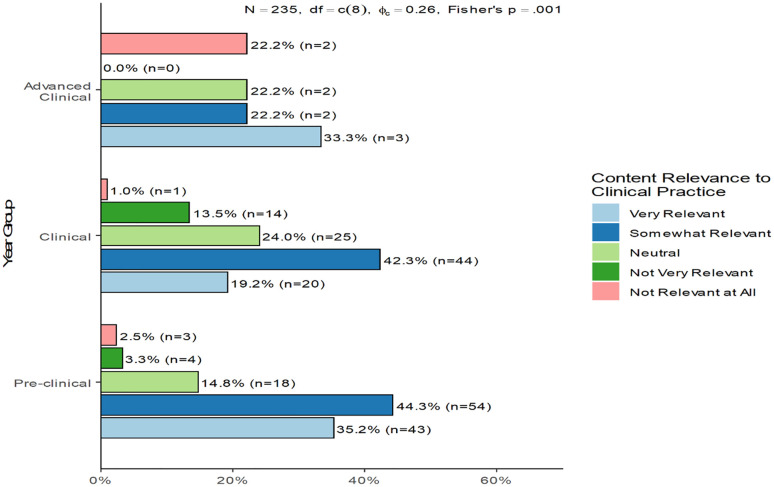
Perceived relevance of histology content to clinical practice across academic year groups.

As illustrated in Fig 3, student perceptions differed significantly across academic stages (Fisher’s p = 0.001, φₐ = 0.26). In the pre-clinical group, the majority of students (44.3%) rated histology content as somewhat relevant to clinical practice, followed by 35.2% who found it very relevant with very few negative responses, indicating strong early appreciation of histology’s foundational value. Among clinical students, somewhat relevant was again the most selected response (42.3%), suggesting moderate but consistent recognition of histology’s applicability during clinical exposure, although a notable proportion (24%) reported a neutral stance. In the advanced clinical group, responses were more evenly distributed. The most common rating was very relevant (33.3%), but an equal 22.2% marked it as somewhat relevant, neutral, or not relevant at all. This fragmentation highlights varying experiences in senior clinical years and possibly a reduced emphasis on histology in advanced rotations.

As shown in Table 5, higher perceived effectiveness of teaching lectures (OR = 1.54, 95% CI: 1.02–2.38, *p* = 0.040), laboratory sessions (OR = 1.56, 95% CI: 1.15–2.17, *p* = 0.006), and integration of histology with clinical courses (OR = 2.33, 95% CI: 1.56–3.57, *p* < 0.001) were all independently and significantly associated with increased odds of positive perception. In contrast, gender (male: OR = 0.47, 95% CI: 0.20–1.08, p = 0.082), academic year group—whether clinical (OR = 0.60, 95% CI: 0.29–1.23, *p* = 0.161) or advanced clinical (OR = 0.48, 95% CI: 0.08–2.84, *p* = 0.419)—histology course completion (OR = 1.10, 95% CI: 0.37–3.11, *p* = 0.853), and perceived course difficulty (OR = 0.83, 95% CI: 0.58–1.18, *p* = 0.307) were not statistically significant predictors of positive perception.

**Table 5:** Logistic regression analysis of factors associated with positive perception of histology.

Predictors	Odds Ratios	95% CI	*p*
**(Intercept)**	204.64	39.35–1320.16	<0.001
**Gender: Male**	0.47	0.20–1.08	0.082
**Year group: Basic**	Ref		
**Year group: Clinical**	0.60	0.29–1.23	0.161
**Year group: Advanced clinical**	0.48	0.08–2.84	0.419
**Histology course completed: Yes vs. No**	1.10	0.37–3.11	0.853
**Course difficulty**	0.83	0.58–1.18	0.307
**Effectiveness of teaching lectures**	1.54	1.02–2.38	0.040
**Effectiveness of teaching labs**	1.56	1.15–2.17	0.006
**Integration effectiveness**	2.33	1.56–3.57	<0.001

The logistic regression analysis identified several factors significantly associated with a positive perception of histology, defined as students rating histology as both very important/important for clinical preparation and reporting being very satisfied/satisfied overall. All ordinal predictors related to teaching quality were included as continuous variables to reflect the odds of positive perception with each 1-point increase in perceived effectiveness.

Table 6 presents Pearson correlation coefficients among four key domains: perception of histology’s value, teaching effectiveness, integration quality, and overall learning experience. All correlations were statistically significant at ***p < 0.001, indicating strong and positive associations among these variables. Notably, perception of value was a moderate positive correlation with teaching effectiveness (r = 0.477) and strongly correlated with integration quality (r = 0.580) and overall experience (r = 0.714), suggesting that students who perceived histology as valuable also rated its integration into the curriculum and their overall learning experience more positively. Similarly, teaching effectiveness was positively correlated with both integration quality (r = 0.539) and overall experience (r = 0.574), highlighting the importance of high-quality instruction in shaping positive student perceptions. Additionally, integration quality was strongly associated with overall experience (r = 0.582), underscoring the vital role of curricular integration in enhancing the educational impact of histology. Moreover, overall experience was significantly associated with all other domains, reinforcing the idea that pedagogical quality and clinical integration are central to enhancing student satisfaction in histology education.

**Table 6:** Pearson correlation matrix among perception domains in histology education.

	Perception of value	Teaching effectiveness	Integration quality	Overall experience
**Perception of value**				
**Teaching effectiveness**	0.477^***^			
**Integration quality**	0.580^***^	0.539^***^		
**Overall experience**	0.714^***^	0.574^***^	0.582^***^	

Computed correlation used Pearson-method with listwise-deletion.

*** *p* < 0.001

## Discussion

This study explored medical students’ perceptions of histology education across academic levels at Imam Mohammad Ibn Saud Islamic University, focusing on its clinical relevance, instructional quality, and curricular integration. The results provide a clear understanding of how students’ views of histology change throughout their medical education and highlight the key teaching and curriculum-related factors that influence these perceptions.

The study included 235 students from different academic stages, most of whom (88%) had completed the histology course, ensuring the validity of their perceptions. While the small proportion of advanced clinical students limits subgroup analyses, the overall distribution enabled comparative analysis across the educational continuum.

Students across all stages generally regarded histology as an important component of their education, with 73.6% rating it as important or very important. However, this perception declined with academic advancement, consistent with previous studies suggesting that basic science subjects are often undervalued in later stages unless explicitly connected to clinical practice [5,6,12,15,20–24]. Consistent with previous findings highlighting the role of basic sciences in supporting diagnostic reasoning, our study showed that 88.5% of students believed histology contributed to their understanding of disease mechanisms, underscoring its continued relevance in clinical decision-making [1].

In terms of relevance to clinical practice, preclinical students showed the most favorable perceptions, while advanced students were more polarized. Satisfaction followed a similar pattern, with higher satisfaction in clinical stages likely reflecting increased awareness of histology’s clinical application. These findings emphasize the need for continuous reinforcement of histology’s relevance throughout the curriculum [25–28].

The current study showed that students overwhelmingly associated histology with pathology (92.3%), followed by oncology (49.8%) and dermatology (43.8%). This supports earlier literature emphasizing histology’s foundational role in tissue-based specialties [5,6,29]. However, its perceived value in radiology, pediatrics, and neurology was markedly lower, suggesting a lack of curricular emphasis in these areas [23,30,31]. The results call for enhanced vertical integration across a broader range of clinical disciplines.

In the current study, students’ perceptions varied significantly across year groups. Preclinical students reported higher ratings for histology’s importance, relevance, and its role in clinical understanding. Clinical students exhibited more neutral and varied perceptions of histology’s importance and relevance, possibly due to limited reinforcement of basic science content during clinical rotations. Advanced clinical students were even more polarized, with higher rates of both strong agreement and disinterest, potentially reflecting diverse specialty interests. This pattern signals missed opportunities for reinforcing basic science relevance during advanced training [31–33].

The current study found that students rated lectures and laboratory sessions positively, with 75.7% and 76.2% describing them as effective or very effective, respectively. Additionally, 80.9% of respondents supported stronger integration of histology with clinical teaching, and 83% expressed a preference for the inclusion of clinical examples, highlighting a clear need for pedagogical reform. These findings are consistent with earlier studies indicating that students highly value histology when it is clearly linked to clinical practice and contextualized within patient care [30,34,35]. Other investigation has also demonstrated that students favor innovative and integrated instructional approaches, particularly those that enhance engagement and clinical relevance [36]. Concerns have been raised about the declining emphasis on histology in modern curricula, despite its acknowledged diagnostic value, reflecting the mixed perceptions observed among clinical and advanced students in our study [37]. Moreover, the integration of digital tools such as virtual microscopy aligns with students’ expressed desire for enhanced visualization and the adoption of modern learning strategies in histology education [38].

This study demonstrated statistically significant variations in students’ perceptions across academic groups. Preclinical students consistently expressed stronger agreement regarding histology’s importance, relevance, and satisfaction. Although clinical students showed slightly lower ratings, they still maintained relatively favorable views particularly in relation to integration and clinical applicability. These findings align with previous research indicating that declining perceptions of basic sciences are often linked to insufficient clinical contextualization as students advance through their training [21]. Similar findings have shown that early-year medical students tend to value histology more highly, while senior students may perceive it as less relevant when not consistently reinforced through clinical exposure [3 5]. A strong association has also been identified between perceived clinical integration and students’ appreciation of histology, indicating that continuous reinforcement across academic stages is essential [3 4]. Conversely, curriculum inconsistency has been identified as a factor contributing to disengagement with histology, particularly in later years of training [3 7].

The regression analysis revealed that the perceived effectiveness of lectures, laboratory sessions, and curricular integration were strong, independent predictors of positive attitudes toward histology. Multiple studies have emphasized that the quality of instructional delivery and curricular integration plays a pivotal role in shaping students’ perceptions of histology. Structured and engaging lectures, combined with interactive laboratory sessions, have been shown to significantly enhance student satisfaction and perceived educational value [39–41]. In particular, teaching strategies that incorporate clinical correlations and promote active learning foster deeper understanding and sustained interest in histological content. Furthermore, strong curricular integration linking histology directly to clinical scenarios has been identified as a key factor in reinforcing its relevance and improving student attitudes [42–44]. These findings align closely with the results of the current study, where lecture quality, lab effectiveness, and integration with clinical teaching emerged as significant predictors of positive perception. Interestingly, demographic factors such as gender and year group were not significant predictors, suggesting that educational design exerts a more influential role than student background.

The Pearson correlation matrices in this study further substantiated the interconnectedness of educational components, providing meaningful insight into how various aspects of histology education are interrelated. Consistent with previous research, students who perceived histology as valuable were significantly more likely to rate teaching effectiveness, clinical relevance, and overall satisfaction highly, emphasizing the importance of instructional alignment and curricular coherence in driving positive learning experiences [43–46]. Satisfaction itself had the highest correlation with applicability, followed by visualization, integration, and disease understanding underscore the need for a multidimensional teaching approach that spans relevance, clarity, integration, and clinical context. Notably, perceived difficulty was not significantly correlated with any positive perception, suggesting that students can accept challenging content if it is delivered meaningfully and engaging manner [47–48]. These findings affirm that when students perceive histology as clinically applicable, clearly visualized, and well-integrated, their satisfaction and perceived value of the course are significantly enhanced.

Taken together, these findings underscore the need to reinforce the clinical relevance of histology consistently across all stages of medical education. Early curricular strategies such as incorporating clinical case examples, problem-based learning (PBL), and virtual microscopy may help bridge the gap between basic science and clinical application. Interdepartmental collaboration between histology and clinical faculty can also ensure that histological principles are revisited and contextualized during clinical rotations, enhancing knowledge retention and relevance.

To support sustained student engagement, institutions should prioritize high-quality teaching, ongoing feedback mechanisms, and continuous curricular integration that explicitly links microscopic anatomy to disease mechanisms and clinical reasoning. Introducing vertically integrated modules that revisit histological concepts in pathology, radiology, and surgery may mitigate the decline in perceived relevance noted in advanced years.

These implications are particularly important within the broader context of curricular reforms in medical education, both locally and globally. While similar studies have been conducted internationally, few have explored student perceptions within Saudi Arabia or institutions that recently adopted outcome-based, integrated curricula. This provides a unique lens to evaluate how educational reforms shape perceptions of foundational sciences. Compared to models in Europe or North America, where histology is often heavily integrated early, our findings emphasize the need for sustained reinforcement in later years. These insights can guide regional and global efforts to optimize histology instruction and maintain its clinical salience throughout medical training.

## Limitations

One notable limitation of this study is the small number of participants in the advanced clinical stage, which may have limited the statistical power of subgroup analyses. Although the overall sample size (n = 235) was adequate for descriptive analysis, it fell short of the originally targeted 400 participants, which may have reduced the ability to detect smaller or subgroup-level effects, particularly in the logistic regression analysis. This limitation introduces a potential risk of Type II error, where genuine differences or associations may not have reached statistical significance due to reduced power. Furthermore, as with all survey-based research, the reliance on self-reported data introduces the potential for response bias, including recall and social desirability bias. Although the overall response rate was methodologically acceptable and supports the validity of the findings, the use of a single-institution sample may limit the generalizability of the results to other medical schools with different curricular designs or student demographics.

Future research should therefore include multi-institutional and longitudinal studies to enhance external validity and explore how evolving clinical exposure influences students’ retention, perception, and application of histological knowledge.

## Conclusion

This study reinforces the essential role of histology in medical education and highlights the importance of its effective integration into clinical teaching. While preclinical students valued histology more highly, perceptions became more varied in later years, reflecting differences in clinical reinforcement. Teaching quality particularly in lectures and labs and curricular integration were key predictors of positive attitudes. Students supported increased clinical contextualization, use of clinical examples, and visualization tools. Notably, course difficulty did not deter engagement when content was delivered meaningfully. Enhancing clinical integration, active learning, and collaboration between basic and clinical faculty—along with digital tools like virtual microscopy can strengthen histology’s relevance and better prepare students for diagnostic practice.

## Supporting information

S1 Table Detailed distribution of students’ perceptions of histology by academic year group. Table S1 provides a detailed breakdown of students’ perceptions of histology by academic stage. Statistically significant trends (*p* < 0.05) were observed across most domains, including perceived importance, clinical relevance, integration with clinical courses, and overall satisfaction. A notable decline in positive responses was seen as students progressed to advanced clinical years. However, views on the use of clinical examples remained consistent (*p* = 0.646), indicating a stable preference for clinically contextualized teaching across all levels.
(DOCX)

S2 Table Correlation matrix of individual variables related to histology perception. This matrix displays Pearson correlation coefficients between various dimensions of histology learning experience. Perception of importance showed strong positive correlations with applicability (r = 0.651), disease understanding (r = 0.631), and satisfaction (r = 0.614), highlighting that students who find histology important also perceive it as applicable and are more satisfied overall. Additionally, integration (r = 0.515), visualization (r = 0.555), and teaching effectiveness in lectures (r = 0.381) and labs (r = 0.348) were positively related to importance. Notably, difficulty showed no significant correlations with other variables, suggesting it may not be a central factor in shaping students’ perceptions. Satisfaction had the strongest correlation with applicability (r = 0.674), followed by importance (r = 0.614), visualization (r = 0.612), integration (r = 0.570), and disease understanding (r = 0.489). These results indicate that students’ satisfaction with histology is mostly influenced by how applicable they find the content, how important they perceive it, and how well the material is visualized and integrated into clinical context.
(DOCX)

## References

[1] Woods NN, Brooks LR, Norman GR. The value of basic science in clinical diagnosis: creating coherence among signs and symptoms. Med Educ. 2005;39(1):107–12. doi: 10.1111/j.1365-2929.2004.02036.x 15612907

[2] Louw G, Eizenberg N, Carmichael SW. The place of anatomy in medical education: AMEE Guide no 41. Med Teach. 2009;31(5):373–86. doi: 10.1080/01421590902825149 19811128

[3] Slivkoff MD, Bahner I, Bonaminio G, Brenneman A, Brooks WS, Chinn C, et al. The Role of Basic Science in 21st Century Medical Education. Med Sci Educ. 2019;29(3):881–3. doi: 10.1007/s40670-019-00760-y 34457556 PMC8368873

[4] Mescher AL. Junqueira’s basic histology: text and atlas. 17 ed. McGraw-Hill Medical; 2023.

[5] Chapman JA, Lee LMJ, Swailes NT. From scope to screen: the evolution of histology education. Adv Exp Med Biol. 2020;1260:75–107. doi: 10.1007/978-3-030-47483-6_5 33211308

[6] Mazzarini M, Falchi M, Bani D, Migliaccio AR. Evolution and new frontiers of histology in bio-medical research. Microsc Res Tech. 2021;84(2):217–37. doi: 10.1002/jemt.23579 32915487 PMC8103384

[7] King TS, Sharma R, Jackson J, Fiebelkorn KR. Clinical Case-Based Image Portfolios in Medical Histopathology. Anat Sci Educ. 2019;12(2):200–9. doi: 10.1002/ase.1794 30118571

[8] Al-Khader A, Obeidat FN, Abu-Shahin N, Khouri NA, Kaddumi EG, Al-Qa’qa’ S, et al. Medical students’ perceptions of pathology and a proposed curricular integration with histology: a future vision of curricular change. Int J Morphol. 2020;38(1):38–42. doi: 10.4067/s0717-95022020000100038

[9] Lakhtakia R. Virtual Microscopy in Undergraduate Pathology Education: An early transformative experience in clinical reasoning. Sultan Qaboos Univ Med J. 2021;21(3):428–35. doi: 10.18295/squmj.4.2021.009 34522409 PMC8407892

[10] Pawlina W. Basic sciences in medical education: why? How? When? Where?. Med Teach. 2009;31(9):787–9. doi: 10.1080/01421590903183803 19811182

[11] Saleh SAK, Adly HM. Integrating basic sciences and clinical practice: a cross-sectional study of UQUMED’s medical education approach. Galician Med J. 2024;31(1). doi: 10.21802/e-gmj2024-a02

[12] Carneiro BD, Pozza DH, Tavares I. Perceptions of medical students towards the role of histology and embryology during curricular review. BMC Med Educ. 2023;23(1):74. doi: 10.1186/s12909-023-04019-4 36717846 PMC9885397

[13] Hortsch M. Histology as a paradigm for a science-based learning experience: Visits by histology education spirits of past, present, and future. Anat Sci Educ. 2023;16(3):372–83. doi: 10.1002/ase.2235 36453080

[14] Custers EJ, ten Cate OT. The history of basic science in medical education. Perspectives on Medical Education. 2011;30(4):311–8. doi: 10.3109/0142159X.2011.558496

[15] Nivala M, Lehtinen E, Helle L, Kronqvist P, Paranko J, Säljö R. Histological knowledge as a predictor of medical students’ performance in diagnostic pathology. Anat Sci Educ. 2013;6(6):361–7. doi: 10.1002/ase.1352 23508971

[16] Escamilla-Sanchez A, López-Villodres JA, Alba-Tercedor C, Ortega-Jiménez MV, Rius-Díaz F, Sanchez-Varo R, et al. Instagram as a tool to improve human histology learning in medical education: descriptive study. JMIR Med Educ. 2025;11:e55861. doi: 10.2196/55861 39970433 PMC11888019

[17] Lam EWM, Chan DWM, Siu FMF, Oluleye BI, Jayasena NS. Pedagogical strategies and critical success factors for enhancing active learning of undergraduate construction and surveying students. Edu Sci. 2024;14(7):703. doi: 10.3390/educsci14070703

[18] Al-Karadsheh O, Abutayyem H, Saidi A, Shqaidef A. Knowledge acquisition and student perceptions of three teaching methods: a randomized trial of live, flipped, and interactive flipped classrooms. BMC Med Educ. 2025;25(1):573. doi: 10.1186/s12909-025-07156-0 40251621 PMC12008932

[19] Alkeheli MF, Akeel IS, Alsulimani OA, Bagazi K, Kabbarah AJ, Othman HI, et al. Evaluation of oral histology curriculum: insights from saudi dental school students-a cross-sectional study. J Med Educ Curric Dev. 2025;12:23821205251327288. doi: 10.1177/23821205251327288 40103581 PMC11915246

[20] Pandey P, Zimitat C. Medical students’ learning of anatomy: memorisation, understanding and visualisation. Med Educ. 2007;41(1):7–14. doi: 10.1111/j.1365-2929.2006.02643.x 17209887

[21] Brauer DG, Ferguson KJ. The integrated curriculum in medical education: AMEE Guide No. 96. Med Teach. 2015;37(4):312–22. doi: 10.3109/0142159X.2014.970998 25319403

[22] Paech D, Giesel FL, Unterhinninghofen R, Schlemmer H-P, Kuner T, Doll S. Cadaver-specific CT scans visualized at the dissection table combined with virtual dissection tables improve learning performance in general gross anatomy. Eur Radiol. 2017;27(5):2153–60. doi: 10.1007/s00330-016-4554-5 27568182

[23] Liu F, Niu P, Yu S, Wei L, He X, Ye J, et al. Investigating students’ perceptions of the medical education environment and learning autonomy in blended learning in pediatrics: a cross-sectional study. BMC Med Educ. 2024;24(1):1464. doi: 10.1186/s12909-024-06490-z 39696173 PMC11654149

[24] Hadad MK, Dehghani MR, Okhovati M, Shafian S. Retention of physiology knowledge among medical students in basic science: a cross-sectional study. BMC Med Educ. 2025;25(1):965. doi: 10.1186/s12909-025-07543-7 40598391 PMC12217992

[25] Muller JH, Jain S, Loeser H, Irby DM. Lessons learned about integrating a medical school curriculum: perceptions of students, faculty and curriculum leaders. Med Educ. 2008;42(8):778–85. doi: 10.1111/j.1365-2923.2008.03110.x 18627445

[26] Almansour M, Abouammoh N, Idris RB, Alsuliman OA, Alhomaidi RA, Alhumud MH, et al. Exploring medical students’ experience of the learning environment: a mixed methods study in Saudi medical college. BMC Med Educ. 2024;24(1):723. doi: 10.1186/s12909-024-05716-4 38961412 PMC11223348

[27] Aslam P, Mushtaq Q, Noor F, Maqbool S, Khan NY, Sarfraz J. The literature review on curriculum implementation problems. JHRR. 2024;4(2):497–501. doi: 10.61919/jhrr.v4i2.844

[28] Sharkas GF, El-Masry R, Abdel-Ghany S, Baz AE, Abou-Elsaad T, Kassab AA, et al. Satisfaction of academic medical staff with integrated medical curriculum: an exploratory multinational survey. BMC Med Educ. 2024;24(1):1483. doi: 10.1186/s12909-024-06468-x 39695623 PMC11656885

[29] Barsoum I, Tawedrous E, Faragalla H, Yousef GM. Histo-genomics: digital pathology at the forefront of precision medicine. Diagnosis (Berl). 2019;6(3):203–12. doi: 10.1515/dx-2018-0064 30827078

[30] Estai M, Bunt S. Best teaching practices in anatomy education: a critical review. Ann Anat. 2016;208:151–7. doi: 10.1016/j.aanat.2016.02.010 26996541

[31] Pusparajah P, Goh BH, Lee L-H, Law JWF, Tan LT-H, Letchumanan V, et al. Integrating the basic and clinical sciences throughout the medical curriculum: contemplating the why, when and how. Prog Drug Discov Biomed Sci. 2022;5(1). doi: 10.36877/pddbs.a0000308

[32] Challa KT, Sayed A, Acharya Y. Modern techniques of teaching and learning in medical education: a descriptive literature review. MedEdPublish (2016). 2021;10:18. doi: 10.15694/mep.2021.000018.1 38486533 PMC10939590

[33] Konar S, Sarkar S, Mondal MK, Aggarwal P. A study based on perception of first phase undergraduate medical students and faculty on integrated teaching in anatomy. Nat J Clin Anat. 2023;12(4):186–90. doi: 10.4103/njca.njca_93_23

[34] Waseem N, Rasheed A, Gill M, Asad A, Shamim MO, Waseem F. The attitudes of medical students towards clinical relevance of histology. PAFMJ. 2021;71(1):351–6. doi: 10.51253/pafmj.v71i1.3756

[35] Teshome D. Attitude and perception of medical students towards histology subject at wollo university, Ethiopia. Adv Med Educ Pract. 2022;13:337–44. doi: 10.2147/AMEP.S359703 35469297 PMC9034839

[36] Taş F. Health sciences students’ viewpoint on innovative approaches in histology course. J Surg Med. 2022;6(12):981–5. doi: 10.28982/josam.7581

[37] Meyer AJ, Chapman JA. A slide into obscurity? The current state of histology education in Australian and Aotearoa New Zealand medical curricula in 2022-2023. Anat Sci Educ. 2024;17(9):1694–705. doi: 10.1002/ase.2518 39407351 PMC11612314

[38] Ambardini RL, Arovah NI, Indra EN. Enhancing histology learning in sports science education through a functional approach and virtual microscopy practical sessions. J Sport Area. 2024;9(3):377–85. doi: 10.25299/sportarea.2024.vol9(3).16552

[39] Gribbin W, Wilson EA, McTaggart S, Hortsch M. Histology education in an integrated, time-restricted medical curriculum: academic outcomes and students’ study adaptations. Anat Sci Educ. 2022;15(4):671–84. doi: 10.1002/ase.2127 34363740

[40] Hortsch M, Girão-Carmona VCC, de Melo Leite ACR, Nikas IP, Koney NK-K, Yohannan DG, et al. Teaching cellular architecture: the global status of histology education. Adv Exp Med Biol. 2023;1431:177–212. doi: 10.1007/978-3-031-36727-4_9 37644293

[41] Hortsch M. Transforming histology students from passive lecture listeners into active lecture learners. Anat Sci Educ. 2024;17(6):1174–82. doi: 10.1002/ase.2463 38816963

[42] Darici D, Flägel K, Sternecker K, Missler M. Transfer of learning in histology: insights from a longitudinal study. Anat Sci Educ. 2024;17(2):274–86. doi: 10.1002/ase.2363 38158384

[43] Souza E Silva R, da Cunha Lima Freire G, Cerqueira GS. The impact of the integration of digital platforms and active teaching strategies (Kahoot!) on the performance of Brazilian medical course students in the discipline of histology. Anat Sci Educ. 2024;17(6):1229–38. doi: 10.1002/ase.2433 38736103

[44] Vieno-Corbett K, Deweyert AM. Celebrating student engagement in an undergraduate histology course: A showcase review. Anat Sci Educ. 2025;18(4):379–85. doi: 10.1002/ase.70011 39991986 PMC11960424

[45] Felszeghy S, Pasonen-Seppänen S, Koskela A, Mahonen A. Student-focused virtual histology education: do new scenarios and digital technology matter?. MedEdPublish. 2017;6:154. doi: 10.15694/mep.2017.000154 38406405 PMC10885289

[46] McTaggart S, Hortsch M. Early practice makes histology masters: the use of a formative assessment quiz to prepare histology learners for a high-stakes final examination. Anat Sci Educ. 2024;17(6):1265–74. doi: 10.1002/ase.2472 38867403

[47] García M, Victory N, Navarro-Sempere A, Segovia Y. Students’ views on difficulties in learning histology. Anat Sci Educ. 2019;12(5):541–9. doi: 10.1002/ase.1838 30378295

[48] Kristoffersen CSM, Ziesler CEØ, Thune NH, Kristensen AT, Sehic A, Utheim TP, et al. Towards a modernized framework of histology teaching to integrate genetics: pedagogical perspectives for oral histology. Genes (Basel). 2025;16(5):512. doi: 10.3390/genes16050512 40428337 PMC12111053

