# Supplementary material for: Perceptions of medical students at Imam Mohammad Ibn Saud Islamic University on histology’s role in clinical course preparation: A cross-sectional study
Source: PLoS One. 2025 Dec 5;20(12):e0337894. doi: 10.1371/journal.pone.0337894 (PMC12680232; doi:10.1371/journal.pone.0337894)
Supplement: S1 Table — Table S1 provides a detailed breakdown of students’ perceptions of histology by academic stage. Statistically significant trends (p < 0.05) were observed across most domains, including perceived importance, clinical relevance, integration with clinical courses, and overall satisfaction. A notable decline in positive responses was seen as students progressed to advanced clinical years. However, views on the use of clinical examples remained consistent (p = 0.646), indicating a stable preference for clinically contextualized teaching across all levels. (DOCX) [file pone.0337894.s001.docx]

**Table S 1. Detailed distribution of students’ perceptions of histology by academic year group**

|  | **Pre-clinical** | **Clinical** | **Advanced Clinical** | **p. overall** | **p. trend** |
| --- | --- | --- | --- | --- | --- |
|  | **N=122** | **N=104** | **N=9** |  |  |
| **Histology importance:** |  |  |  | 0.001 | <0.001 |
| **Very important** | 61 (50.0%) | 26 (25.0%) | 3 (33.3%) |  |  |
| **Important** | 39 (32.0%) | 43 (41.3%) | 1 (11.1%) |  |  |
| **Neutral** | 17 (13.9%) | 21 (20.2%) | 3 (33.3%) |  |  |
| **Not important** | 2 (1.6%) | 10 (9.6%) | 2 (22.2%) |  |  |
| **Not important at all** | 3 (2.5%) | 4 (3.9%) | 0 (0.00%) |  |  |
| **Histology–clinical relevance:** |  |  |  | 0.017 | 0.025 |
| **Very strong** | 49 (40.2%) | 23 (22.1%) | 5 (55.6%) |  |  |
| **strong** | 55 (45.1%) | 55 (52.9%) | 1 (11.1%) |  |  |
| **moderate** | 12 (9.8%) | 17 (16.3%) | 2 (22.2%) |  |  |
| **weak** | 5 (4.1%) | 8 (7.7%) | 1 (11.1%) |  |  |
| **No connection** | 1 (0.8%) | 1 (0.1%) | 0 (0.00%) |  |  |
| **Histology aids clinical understanding:** |  |  |  | 0.088 | 0.006 |
| **Yes, significantly** | 70 (57.4%) | 47 (45.2%) | 2 (22.2%) |  |  |
| **Yes, somewhat** | 43 (35.2%) | 41 (39.4%) | 5 (55.6%) |  |  |
| **No, not really** | 6 (4.9%) | 12 (11.5%) | 1 (11.1%) |  |  |
| **No, not at all** | 3 (2.45%) | 4 (3.9%) | 1 (11.1%) |  |  |
| **Content relevance to clinical practice:** |  |  |  | < 0.001 | 0.001 |
| **Very relevant** | 43 (35.2%) | 20 (19.2%) | 3 (33.3%) |  |  |
| **Somewhat relevant** | 54 (44.3%) | 44 (42.3%) | 2 (22.2%) |  |  |
| **Neutral** | 18 (14.8%) | 25 (24.0%) | 2 (22.2%) |  |  |
| **Not very relevant** | 4 (3.3%) | 14 (13.5%) | 0 (0.00%) |  |  |
| **Not relevant at all** | 3 (2.5%) | 1 (0.1%) | 2 (22.2%) |  |  |
| **Integration with clinical courses:** |  |  |  | . | <0.001 |
| **Very well integrated** | 46 (37.7%) | 20 (19.2%) | 3 (33.3%) |  |  |
| **Well integrated** | 52 (42.6%) | 33 (31.7%) | 1 (11.1%) |  |  |
| **Neutral** | 20 (16.4%) | 38 (36.5%) | 2 (22.2%) |  |  |
| **Poorly integrated** | 3 (2.5%) | 13 (12.5%) | 3 (33.3%) |  |  |
| **Not integrated at all** | 1 (0.8%) | 0 (0.00%) | 0 (0.00%) |  |  |
| **Preference for clinical examples:** |  |  |  | 0.646 | 0.800 |
| **Yes, definitely** | 60 (49.2%) | 49 (47.1%) | 4 (44.4%) |  |  |
| **Yes, somewhat** | 40 (32.8%) | 40 (38.5%) | 2 (22.2%) |  |  |
| **No, prefer basic** | 19 (15.6%) | 12 (11.5%) | 3 (33.3%) |  |  |
| **Not sure** | 3 (2.5%) | 3 (2.9%) | 0 (0.00%) |  |  |
| **Overall course satisfaction:** |  |  |  | . | <0.001 |
| **Very satisfied** | 53 (43.4%) | 14 (13.5%) | 3 (33.3%) |  |  |
| **Satisfied** | 50 (41.0%) | 43 (41.3%) | 2 (22.2%) |  |  |
| **Neutral** | 13 (10.7%) | 34 (32.7%) | 2 (22.2%) |  |  |
| **Dissatisfied** | 5 (4.1%) | 11 (10.6%) | 1 (11.1%) |  |  |
| **Very dissatisfied** | 1 (0.8%) | 2 (1.9%) | 1 (11.1%) |  |  |
