# Supplementary material for: Perceptions of medical students at Imam Mohammad Ibn Saud Islamic University on histology’s role in clinical course preparation: A cross-sectional study
Source: PLoS One. 2025 Dec 5;20(12):e0337894. doi: 10.1371/journal.pone.0337894 (PMC12680232; doi:10.1371/journal.pone.0337894)
Supplement: S2 Table — This matrix displays Pearson correlation coefficients between various dimensions of histology learning experience. Perception of importance showed strong positive correlations with applicability (r = 0.651), disease understanding (r = 0.631), and satisfaction (r = 0.614), highlighting that students who find histology important also perceive it as applicable and are more satisfied overall. Additionally, integration (r = 0.515), visualization (r = 0.555), and teaching effectiveness in lectures (r = 0.381) and labs (r = 0.348) were positively related to importance. Notably, difficulty showed no significant correlations with other variables, suggesting it may not be a central factor in shaping students’ perceptions. Satisfaction had the strongest correlation with applicability (r = 0.674), followed by importance (r = 0.614), visualization (r = 0.612), integration (r = 0.570), and disease understanding (r = 0.489). These results indicate that students’ satisfaction with histology is mostly influenced by how applicable they find the content, how important they perceive it, and how well the material is visualized and integrated into clinical context. (DOCX) [file pone.0337894.s002.docx]

|  | **Importance** | **Clinical relation** | **Disease understanding** | **Applicability** | **Difficulty** | **Lectures** | **Laboratory** | **Integration** | **Visualization** | **Satisfaction** |
| --- | --- | --- | --- | --- | --- | --- | --- | --- | --- | --- |
| **Importance** |  |  |  |  |  |  |  |  |  |  |
| **Clinical relation** | 0.554^***^ |  |  |  |  |  |  |  |  |  |
| **Disease understanding** | 0.631^***^ | 0.500^***^ |  |  |  |  |  |  |  |  |
| **Applicability** | 0.651^***^ | 0.549^***^ | 0.559^***^ |  |  |  |  |  |  |  |
| **Difficulty** | -0.038 | 0.007 | -0.001 | -0.002 |  |  |  |  |  |  |
| **Lectures** | 0.381^***^ | 0.268^***^ | 0.283^***^ | 0.340^***^ | -0.075 |  |  |  |  |  |
| **Laboratory** | 0.348^***^ | 0.307^***^ | 0.308^***^ | 0.326^***^ | 0.052 | 0.329^***^ |  |  |  |  |
| **Integration** | 0.515^***^ | 0.398^***^ | 0.429^***^ | 0.555^***^ | -0.059 | 0.412^***^ | 0.464^***^ |  |  |  |
| **Visualization** | 0.555^***^ | 0.415^***^ | 0.490^***^ | 0.534^***^ | -0.104 | 0.355^***^ | 0.416^***^ | 0.471^***^ |  |  |
| **Satisfaction** | 0.614^***^ | 0.417^***^ | 0.489^***^ | 0.674^***^ | -0.096 | 0.409^***^ | 0.485^***^ | 0.570^***^ | 0.612^***^ |  |
| Computed correlation used Pearson-method with listwise-deletion.  Note: * ** Correlation is statistically significant at *p* < 0.001 | | | | | | | | | | |

**Table S 2. Correlation matrix of individual variables related to histology perception**
